# A Review on the Nonpharmacological Therapy of Traditional Chinese Medicine with Antihypertensive Effects

**DOI:** 10.1155/2019/1317842

**Published:** 2019-01-02

**Authors:** Hua Fan, Feng Lu, Ailing Yang, Yidan Dong, Ping Liu, Youhua Wang

**Affiliations:** ^1^Longhua Hospital, Shanghai University of Traditional Chinese Medicine, China; ^2^The Affiliated Hospital of Shandong University of TCM, Shandong Provincial Hospital of Traditional Chinese Medicine, China

## Abstract

Hypertension is a global health concern. Although the pharmacological treatment has obvious antihypertensive effects, there are still some limitations on management of hypertension by drug therapy alone. In recent years, the nonpharmacological therapy of traditional Chinese medicine (TCM) has gradually become an important mean to help the prevention and treatment of hypertension in some Eastern countries. In this review, the nonpharmacological TCM therapies, including acupuncture, tuina, Tai Chi, and auricular-plaster, are covered along with the mechanism.

## 1. Introduction

Hypertension is one of the most important risk factors for heart disease, stroke, chronic kidney disease and so on [1]. Globally, hypertension is responsible for at least 45% of deaths due to heart disease and 51% of deaths due to stroke [2]. In 2017, American Heart Association (AHA) released the new version of the American Guidelines for Hypertension, in which hypertension is defined as blood pressure (BP) ≥130/80 mmHg [3]. Under these circumstances, more adult population will be affected, and hypertension is becoming a more and more serious medical and public health issue. In current treatments for hypertension, there are several classes of antihypertensive drugs and strategies to modify the effect of adverse lifestyles on BP. Although these have made great progress in targeting hypertension, for which a number of approaches have been developed, only about a quarter of patients are receiving adequate treatment.

Pharmacotherapy can effectively control the blood pressure. However, it may be associated with potential adverse events, including hepatic and renal dysfunction. There are still some other deficiencies in drug treatment. For example, prehypertension and stage I hypertension alongside mild hypertension drugs are recommended for intensive lifestyle management rather than antihypertensive drugs for their initial BP lowering. The effect of drugs in the treatment of refractory hypertension is still not satisfactory, and the economic burden of long-term medication may cause lack of compliance to the poor economic conditions patients [4]. Therefore, more and more people have turned to focus on complementary and alternative medicine (CAM) for treating essential hypertension (EH).

Traditional Chinese medicine (TCM) includes many kinds of nonpharmacological interventions, such as acupuncture, tuina, and qigong. They can improve related symptoms of diseases through external stimuli. The American College of Physicians recommended noninvasive pharmacologic and nonpharmacologic treatments such as acupuncture and tuina as first-line treatment for low back pain [5]. In fact, TCM nonpharmacological interventions have a positive effect on the adjuvant treatment of disease. Firstly, they avoid the direct toxic effect of the drugs on the organs because of vitro stimuli. Secondly, some studies [6–10] showed that they can better control the blood pressure in patients with early hypertension. It puts forward good directions for the risk of taking medicine for early hypertension. Thirdly, they can markedly improve the symptoms of hypertension, such as severe headache, dizziness, and fatigue. For some patients with refractory hypertension or serious related symptoms, the quality of life can be improved and the pain can be reduced by TCM nonpharmacological interventions [11–14]. Last but not least, they belong to a low cost treatment. Some acupoint massage or exercise can be carried out by patients themselves paying no money. This review summarizes the methods of TCM nonpharmacological interventions on EH, along with the mechanism.

## 2. Acupuncture

As a famous form of traditional Chinese medicine, acupuncture is one of the oldest and most commonly used forms of alternative medicine. It is a method for restoring balance of qi, the life force that circulates throughout the body in energy pathways that are called meridians, by stimulating specific points over the surface of body known as acupuncture points or acupoints [15]. In recent years, as complementary medicine interventions, acupuncture is increasingly valued by people and widely used in the clinic, as well as being applied in the United States and Europe. It is said that acupuncture can treat a number of diseases, including those related to the cardiovascular system [16], such as hypertension [17]. So it could be used on patients who want to avoid drug therapy or as an alternative option to reduce dosages of antihypertensive agents.

Some systematic reviews and studies [6, 17–19] found that acupuncture might lower blood pressure in prehypertension and stage I hypertension. Acupuncture could reduce blood pressure by regulating the nervous system. Some acupoints could reduce blood pressure by modulating sympathetic nerves [20–23]. Tan Ying-ying et al. [24] established hypertension model with 45 male SD rats and found that electroacupuncture (EA) stimulation of “Quchi” (LI 11) can downregulate arterial blood pressure and sympathetic nerve activity and increase the baroreflex sensitivity in hypertension rats, which may be related to its effects in downregulating p47 phagocyte oxidase mRNA and protein expression in the RVML. One of the antihypertensive mechanisms of acupuncture at Taichong (LR3) is also via the regulation of renal sympathetic activity and ss-ARs [25]. In addition, acupuncture can also stimulate the rostral ventrolateral medulla (RVLM) to regulate blood pressure. The study of Wang Xue-Rui et al. [26] found that acupuncture decreases high blood pressure and nicotinamide adenine dinucleotide phosphate oxidase in the RVLM of spontaneously hypertensive rats. Oxidative stress in the RVLM, where the sympathetic nervous control center is located, contributes to neural mechanisms of hypertension. So the mitogen-activated protein kinases and the sciatic nerve are involved in the mechanism of acupuncture's amelioration of hypertension.

Besides regulating nervous system, acupuncture can also improve hypertension through various ways. For example, EA could significantly reduce markers of cardiac hypertrophy and apoptosis, as well as elevated expression of antioxidant enzymes including superoxide dismutase-1 (SOD1) in SHRs [27]. Sin Bond Leung et al. [28] found that the effects of acupuncture in treating hypertension were associated with reduced oxidative stress, increased nitric oxide bioavailability, and endothelial function in SHRs.

## 3. Tuina

Tuina is based on the theory of internal organs and meridians of TCM. It is pressed on specific parts of the body surface to regulate the body's physiological and pathological conditions, to achieve the purpose of physiotherapy. Modern studies indicate that tuina manipulations modulate BP by the following four aspects [29]. Firstly, it regulates the nervous system, activating vagus, inhibiting sympathetic nerves, and adjusting autonomic nerves in both ways, and further dilating capillaries and reducing the resistance of peripheral blood vessels [30]. Secondly, the manipulations can relax major muscle groups, release vascular spasm, increase vascular bed, and improve blood flow in peripheral vessels [31]. Thirdly, it stimulates the carotid baroreceptor to excite the vagus, reduce heart rate and pumping blood volume, inhibit vasomotor neurons, dilate blood vessels, and decrease the resistance of peripheral blood vessels [32]. Fourthly, the manipulations can also reduce symptoms such as pressure and anxiety [33, 34].

Shen Zhi-fang [35] et al. treated 40 patients with grade 1-2 hypertension and observed that the tuina manipulations of kidney-tonifying blood-circulating and collaterals-unblocking could produce a significant effect on antihypertension and improving blood pressure variability, and it also could effectively reduce the target organ damage in hypertension and benefit the prognosis. In addition, tuina can also improve sleep quality [36, 37] in middle-aged Women and old people with hypertension.

## 4. Qigong

Qigong is an ancient Chinese healing art, involving meditation, controlled breathing, and movement exercises that date back thousands of years, and is believed to be based on TCM. Qigong includes two concepts: qi, the vital energy of the body and gong, the training or cultivation of qi. Medical qigong is divided into internal and external components [38]. Internal qigong consists of exercises, including breathing, meditation, focus of intention, and rhythmical movements, while external qigong is performed by a trained practitioner in order to deliver qi energy to the patient. One possible explanation for the beneficial effects of qigong exercise is increased healthy flow of qi, blood, and fluid throughout the body by repetitive movements to relieve pathological stagnation and regulate the function of meridians and visceral organs. Many potential beneficial effects have been found on various disorders [39], including cardiovascular disorders, rheumatoid arthritis, asthma, and cancers. And qigong has also been studied as an alternative therapy for controlling blood pressure.

For example, Ba duan jin qigong exercise [40–43] may have the potential to improve systolic blood pressure (SBP), diastolic blood pressure (DBP), high-density lipoprotein cholesterol (HDL-C), low-density lipoprotein cholesterol (LDL-C), total cholesterol (TC), triglycerides, fasting glucose, serum NO, and plasma endothelin-1 and then improve cardiovascular function and decrease BP in older adults with hypertension. Randomized clinical trials [44] also showed that the possible mechanisms by which qigong could reduce BP in hypertensive patients may include modulation of the sympathoadrenal medulla function, while the lowering of plasma norepinephrine and epinephrine levels can reduce sympathetic nervous system activity. Therefore, a decrease in catecholamine levels through modulation of the sympathetic adreno medullary function could be the mechanism underlying the observed reduction in blood pressure.

## 5. Tai Chi

Tai Chi also originates from China. It is a therapy which evolved from martial arts. Tai Chi [45] mainly comprises three basic components, movement, deep breathing, and meditation. The movement of this exercise is slow, fluid, and continuous. They need body, breathing, and heart to achieve a balanced state. In this way, the essence, qi and spirit of the body can form a benign cycle. Doing this exercise [46] can relax the body and mind, strengthen the joints, improve joint mobility, and boost the immune system. The slow movements [47] between different postures that are normally held for short periods of time represent physical stimuli, which affect the cardiovascular and muscular systems.

Tai Chi exercise [48, 49] had shown positive effects on patients with many chronic diseases. Many systematic reviews [50–53] of Tai Chi also showed that this sport has beneficial effects on BP of patients with hypertension. And a community-based study [54], which include 266 patients with hypertension, found that a Tai Chi program to improve hypertension in older adults is effective in reducing blood pressure and body mass index, maintaining normal renal function and improving physical health of health-related quality of life. The study of PAN Xiaogui [55] et al. found that SBP, DBP, and mean arterial pressure levels can significantly reduce in 24 EH patients who practice Tai Chi exercise, compared with 16 patients without exercise. And the reduced blood pressure may be correlated with increased plasma levels of gaseous signal molecules, such as endogenous NO, CO, and H_2_S, which are involved in blood pressure regulation.

## 6. Auricular-Plaster

Auricular-plaster has been utilized in the treatment of diseases for thousands of years. Dr. Paul Nogier firstly originated the concept of an inverted fetus map on the external ear. A present study [56] indicates that the auricular-plaster plays a role in vagal activity of autonomic functions of cardiovascular, respiratory, and gastrointestinal systems. So auricular-plaster is also proposed to prevent hypertension via vagal regulation.

Some meta-analysis trials [57–59] show that auricular pressure therapy is safe and effective for EH. Yang Xiaolin [12] et al. divided ninety patients with primary hypertension into medication group and combination group. After 4 weeks of treatment, they found that auricular point sticking combined with acupuncture for primary hypertension is better than captopril for the improvement of 24 h ambulatory blood pressure, Ang II level and creatinine level, and can improve dizzyness, palpitation, and other clinical symptoms. It [60, 61] can also be applied at the acupoints of Shenmen, sympathesis, kidney, liver, heart, and subcortex to improve physical pain and reduce target organ damage for hypertensive patients.

## 7. Acupoint Catgut Embedding

Acupoint catgut embedding [62] refers to setting absorbable catgut into the acupoint, by stimulating meridians, balancing yin and yang, and harmonizing qi and blood, to achieve the purpose of adjusting viscera and treating diseases. Modern medical theory holds that the inflammatory reaction occurred in the local package by catgut to produce continuous stimulation on acupoints. By stimulating the acupuncture point, it can restore the regulation function of central nervous system and endocrine body fluid, relive blood vessel spasm, and steady the blood pressure.

Clinical trials [11, 63–65] showed that acupoint catgut embedding therapy can better improve the symptoms and blood pressure of patients with hypertension. Li Ling [66] et al. treated 70 prehypertensive patients with acupoint catgut embedding and found that this treatment also can effectively downregulate the total cholesterol, triglyceride, and low-density lipoprotein cholesterol, upregulate the high-density lipoprotein cholesterol, and reduce weight. The mechanism [67–69] of acupoint catgut embedding may be sustained stimulation of acupoints, which regulate the excitability of the central nervous system, decrease the content of renin-angiotensin-aldosterone, improve the microvascular circulation and relaxation, and finally regulate the arterial blood pressure reaching a new balance.

## 8. Chinese Medicine Foot Bath

TCM believes that the feet have many meridians and acupoints connected with viscera. For example, high blood pressure is also located at the toes, and long-term compression can make the body in the dynamic balance of qi and blood [70]. Chinese medicine foot bath therapy [71, 72] has the following three main effects on hypertension. (1) Improving the blood circulation of the whole body and regulating the autonomic nerve. (2) Reducing blood viscosity. (3) Promoting metabolism. It has the dual effect of the water temperature and medicine liquid fumigation: this therapy, combined with the effect of drug heat and drug penetration [73], can increase the inhibition process of cerebral cortex through the mechanism of nerve reflex and body fluid, so as to regulate the action of the subcortical vascular motor center, reduce the tension of peripheral arteriole, and assist in the treatment of hypertension [74].

Some clinical observations [75, 76] showed that, compared with common therapy, treatment with Chinese medicine foot bath can perfect better in reducing systolic and diastolic pressure, and improving symptoms like vertigo, headache, palpitation, and shortness of breath. Huang Yao [77] et al. intervened in 152 patients with prehypertension and primary grade 1 hypertension by Zhu Liangchun's foot bath recipe and found that the clinical curative effect is promising. Particularly, it has obvious effect on decreasing DBP. Cui Yi [78] et al. observed 140 patients with different stages of hypertension and found that Chinese medicine foot bath has significant effect on mild, moderate, and severe hypertension.

## 9. Other Therapies

In fact, there are still other therapies for EH in folk, such as Chinese medicine pillow, which worked though volatility of herbs [79–81], and Chinese umbilical, which absorbed drugs into the blood through navel [82, 83] and special diet. Some studies showed that these therapies also can relieve hypertension and its related symptoms of patients in varying degrees. But these researches are only in the stage of clinical observation and not going on deeper mechanism study.

In summary, the abundant nonpharmacological therapies could not only enhance drug effect but also have many other advantages, such as patient's willingness to accept, cheapness, no toxic side effects and so on. However, the basic research on TCM nonpharmacological therapies for hypertension is still inadequate based on the existing literature. Firstly, the regulations of indications or contraindications of different treatments are still unclear. For example, exercise therapy may be suitable for critical hypertension and stages I and II hypertension, but not for the stage III hypertension, or other serious complications. Therefore, different types of hypertension patients should be in accordance with the doctor's exercise prescription, to choose the appropriate sports and sports intensity, and pay attention to the safety of sports. Secondly, the effectiveness of various nonpharmacological interventions has yet to be further studied. There are many reports on the treatment, but the mechanism of nonpharmacological interventions is still not clear. What is more, we can see the improvement of the risk factors and target organ damage are mentioned in many articles; however, they need to be further studied and discussed in the future.

As we know, the ultimate goal of hypertension treatment should be to reduce the incidence and mortality of heart, brain, and kidney complications. Although more and more people tend to use TCM nonpharmacological interventions, well-designed preclinical and clinical trials on the potential synergistic and adverse side effects of the therapies, as well as their mechanisms, are warranted. And TCM nonpharmacological interventions should be selected under the guidance of doctors.
